# Medical Opinion and Movement

**Published:** 1906-06-16

**Authors:** 


### Medical Opinion and Movement.

The opposition of the Central Hospital Council for London to the State registration of nurses which has been brought to the notice of the British Medical Association appears to be carrying weight with the profession. The Glasgow and West of Scotland branch having considered the methods of registration proposed by the Select Committee of the House of Commons, in conjunction with these objections, decided not to approve the recommendations of the Select Committee.

The invitation of Messrs. Armour and Company to the members of the British Medical Association to visit their packing establishments and examine into the process followed is causing some discussion in medical circles. This invitation is reported to be accompanied by an offer to pay the expenses of all the members who consent to visit Messrs. Armour's establishments, an offer which, if accepted, would destroy any useful effect which such an inspection could have upon Messrs. Armour's business. The President of the United States has wisely taken the view that it is very difficult to deal with some of the manifest railway evils which are crying for reform, if he accepts free transit from the railways when visiting the different States officially. Mr. Roosevelt's view will no doubt be the view which will be taken by the Council and members of the British Medical Association in regard to the Armour offer of free quarters and fares to those who undertake to inspect and report upon their packing establishments.

The growth of consumption sanatoria cannot be regarded with indifference by the medical profession. At best such developments are limited in their effect, seeing that the majority of the patients cannot afford to pay the weekly charges, so that tuberculous patients are unable to avail themselves in any numbers, if at all, of this accommodation. What the public needs is an active propaganda based upon an authoritative statement and scientific facts, which will show relatively humble people how they can safely retain a case of tuberculous disease in the family, and provide a chalet or open-air residence at small cost in their garden, where, as in very many cases, the family resides in a healthy suburb or in the country. The Open-air League, which has an advisory committee of which Dr. Goodhart and three other Fellows of the Royal College of Physicians are members, might usefully add to its objects that of undertaking the propaganda just referred to. It would further do a public service if it were to make it clear in all its documents that under proper precautions the senseless panic which sometimes affects families who fear infection, is unjustifiable in the case of everybody who has any pretension to the possession of common sense.

The opening of the King Edward VII. Sanatorium near Midhurst, on the Sussex Hills, by the King on Wednesday, June 13th, was an occasion full of interest to members of the medical profession. The building is imposing, and constitutes the largest of the costly structures which have been erected in recent years in response to a demand which has been so relatively hurried and imperative as to lead to an outpouring of money, much of which has probably been wasted. This medical science will probably demonstrate in the near future. First of all, many of those who have given the greatest attention to this class of building and method of treatment are practically unanimous in declaring that costly buildings of a permanent character are not desirable or necessary, and that on the whole it is best to avoid them when making provision for the open-air treatment of tuberculous cases. There is, too, a growing hope in the profession that medical science may so develop as to render it possible by treatment to effectively deal with this class of cases, and so to supersede the open-air treatment altogether. The latter form of treatment will probably prove in a few years to have been but tentative, in which case the costly buildings referred to may cease to be used for tuberculous cases, when the position would become anything but satisfactory from an economical point of view. Should experience confirm the views held by Sir William MacEwen in regard to the surgical treatment of certain conditions of the lung, the changes which are likely to result may prove momentous in the history of medicine and of the first importance to the patients and the methods of treatment which have hitherto prevailed.

The profession will welcome the fact that the King's Sanatorium will provide much-needed accommodation for persons of the middle and lower middle classes, whose slender means have rendered it impossible for them to defray the cost of sanatorium treatment under existing conditions. Unfortunately patients who have obtained the best results from the open-air treatment very frequently suffer from relapses on their return to their former life and occupations, owing to the fact that the necessity for continuing this treatment and discipline has not been sufficiently understood or insisted upon in the past. There are few such homes which are not provided with some garden space at any rate, where it should be feasible at very little cost to erect a chalet in which the convalescent patient could continuously live under conditions best calculated to promote health and physical vigour. This point has been too much overlooked in the past, and we hope that the attention which the King's presence at Midhurst must attract to tuberculosis may lead the health authorities, and all who have it in their power to influence the after-care of convalescents from this disease, to concert measures in the direction we have indicated. The influence of the profession, too, is urgently called for in the direction of minimising the unreasoning panic which has affected certain families in the past where a case of tuberculosis has occurred, and which has led to several cases of appalling cruelty on the part of the patients' friends, which are far from creditable to those concerned.

